# Molecular and Structural Basis of DNA Sensors in Antiviral Innate Immunity

**DOI:** 10.3389/fimmu.2020.613039

**Published:** 2020-11-30

**Authors:** Ayesha Zahid, Hazrat Ismail, Bofeng Li, Tengchuan Jin

**Affiliations:** ^1^ Department of Obstetrics and Gynecology, The First Affiliated Hospital of USTC, Division of Life Sciences and Medicine, University of Science and Technology of China, Hefei, China; ^2^ Hefei National Laboratory for Physical Sciences at Microscale, the CAS Key Laboratory of Innate Immunity and Chronic Disease, School of Basic Medical Sciences, Division of Life Sciences and Medicine, University of Science and Technology of China, Hefei, China; ^3^ MOE Key Laboratory for Cellular Dynamics & Anhui Key Laboratory for Chemical Biology, CAS Center for Excellence in Molecular Cell Science, Hefei National Science Center for Physical Sciences at Microscale & University of Science and Technology of China, Hefei, China; ^4^ Department of Medical Oncology, The First Affiliated Hospital of USTC, Division of Life Sciences and Medicine, University of Science and Technology of China, Hefei, China; ^5^ CAS Center for Excellence in Molecular Cell Science, Chinese Academy of Science, Shanghai, China

**Keywords:** DNA sensors, pattern-recognition receptors, cyclic GMP-AMP synthase, STING, Toll-like receptor 9, interferon-gamma inducible 16, absent in melanoma 2, RNA polymerase III

## Abstract

DNA viruses are a source of great morbidity and mortality throughout the world by causing many diseases; thus, we need substantial knowledge regarding viral pathogenesis and the host’s antiviral immune responses to devise better preventive and therapeutic strategies. The innate immune system utilizes numerous germ-line encoded receptors called pattern-recognition receptors (PRRs) to detect various pathogen-associated molecular patterns (PAMPs) such as viral nucleic acids, ultimately resulting in antiviral immune responses in the form of proinflammatory cytokines and type I interferons. The immune-stimulatory role of DNA is known for a long time; however, DNA sensing ability of the innate immune system was unraveled only recently. At present, multiple DNA sensors have been proposed, and most of them use STING as a key adaptor protein to exert antiviral immune responses. In this review, we aim to provide molecular and structural underpinnings on endosomal DNA sensor Toll-like receptor 9 (TLR9) and multiple cytosolic DNA sensors including cyclic GMP-AMP synthase (cGAS), interferon-gamma inducible 16 (IFI16), absent in melanoma 2 (AIM2), and DNA-dependent activator of IRFs (DAI) to provide new insights on their signaling mechanisms and physiological relevance. We have also addressed less well-understood DNA sensors such as DEAD-box helicase DDX41, RNA polymerase III (RNA pol III), DNA-dependent protein kinase (DNA-PK), and meiotic recombination 11 homolog A (MRE11). By comprehensive understanding of molecular and structural aspects of DNA-sensing antiviral innate immune signaling pathways, potential new targets for viral and autoimmune diseases can be identified.

## Introduction

Viruses are a threat to humans since ancient times; therefore, many mechanisms exist in the human body to cope with viral infections. A tremendous amount of resources is utilized worldwide to control the spread of viral infections because such infections pose a huge burden to the health sector by resulting in life-threatening diseases. The innate immune system is the body’s first line of defense against pathogenic microbes and is essential in conferring antiviral immune responses, which ultimately lead to the pathogen clearance. Numerous innate immune receptors named pattern-recognition receptors (PRRs) are present at the cell surface or within the cells, which are employed by the innate immune defense to detect conserved structural features of the pathogens called pathogen-associated molecular patterns (PAMPs) (1). In the case of viruses, PAMPs include viral genomic material, surface structures such as glycoproteins, capsids, and replication products. Millions of years of evolution have evolved PRRs substantially in three ways: (i) they not only control the infection but also induce cellular senescence (2); (ii) they operate at cellular intrinsic levels and meanwhile are associated with cellular machinery so that a danger signal can be relayed to the local microenvironment when necessary (3, 4); and (iii) they have obtained the capability to detect the presence of non-compartmentalized host nucleic acids (5, 6). Hence, mammalian cells can utilize PPRs to execute a response to the dangerous build-up of endogenous or exogenous nucleic acids. Multiple receptors can recognize a single virus, and one receptor may target different viruses (7). Pathogen-derived nucleic acids as single-stranded (ss) or double-stranded (ds) DNA and RNA serve as the most potent PAMPs that derive antiviral responses that are fundamental for the induction of resulting acquired immunity (8). Over the last decade, several nucleic acid sensors, including members of toll-like receptors (TLRs), RIG-I like receptors (RLRs), NOD-like receptors (NLRs) families, and cyclic GMP-AMP synthase protein families have been identified. Signaling pathways that result in the synthesis of interferons, inflammatory cytokines, and chemokines are triggered by the activation of such receptors and lead to anti-viral inflammatory and cell-mediated immune responses (9–11). Two paradigmatic cytosolic nucleic acid sensing pathways in mammalian cells include the cGAS-STING (cyclic GMP-AMP synthase-stimulator of interferon genes) pathway and RLR-MAVS (RIG-I like receptor-mitochondrial antiviral signaling protein) pathway, which sense cytosolic DNA and RNA respectively (12). **Table 1** lists DNA sensors that detect the nucleic acids of various viruses, bacteria and fungi.

**Table 1:** A list of DNA sensors which detect the nucleic acids of various viruses, bacteria and fungi.

Pathogen	Genome	Family	Primary host (s)	DNA Sensor(s)	References
Herpes simplex virus (HSV)	dsDNA	Herpesviridae	Human	TLR9, RNA pol III, IFI16, DAI, DHX9, DHX36, DDX41, MRE-11, cGAS	(13–23)
Varicella zoster virus (VZV)	dsDNA	Herpesviridae	Human	TLR9, RNA pol III	(24, 25)
Human cytomegalovirus (HCMV)	dsDNA	Herpesviridae	Human	TLR9, DAI, cGAS	(15, 26, 27)
Murine cytomegalovirus (MCMV)	dsDNA	Herpesviridae	Mouse	AIM2	(28)
Epstein-Barr virus (EBV)	dsDNA	Herpesviridae	Human	TLR9, RNA pol III, cGAS, IFI16	(16, 29–31)
Vaccinia virus (VV)	dsDNA	Poxviridae	Unknown	TLR9, AIM2, DNA-PK, cGAS	(15, 28, 32, 33)
Kaposi’s sarcoma-associated herpesvirus (KSHV)	dsDNA	Herpesviridae	Human	TLR9, IFI16, cGAS	(17, 32, 34)
Adenovirus (AdV)	dsDNA	Adenoviridae	Unknown	TLR9, DDX41, cGAS	(15, 18, 35)
Human papilloma virus (HPV)	dsDNA	Herpesviridae	Human	TLR9, cGAS	(15, 36)
Murine gammaherpesvirus 68 (MHV68)	dsDNA	Herpesviridae	Rodent	cGAS	(15)
Ectromelia virus (ECTV)	dsDNA	Poxviridae	Mouse	TLR9	(37)
Human immunodeficiency virus (HIV)	ssRNA	Retroviridae	Human	cGAS, TLR9	(38, 39)
Simian immunodeficiency virus (SIV)	ssRNA	Retroviridae	Non-human primates	cGAS	(39)
Murine leukemia virus (MLV)	ssRNA	Retroviridae	Mouse	cGAS	(39)
West Nile virus (WNV)	ssRNA	Flaviviridae	Human	cGAS	(40)
Dengue virus (DENV)	ssRNA	Flaviviridae	Human	cGAS	(40)
Vesicular stomatitis virus (VSV)	ssRNA	Rhabdoviridae	Cattle, horses, and swine	cGAS, DHX60	(41, 42)
Influenza A virus	dsRNA	Orthomyxoviridae	Birds and mammals	DHX36, DHX9	(43, 44)
*Neisseria meningitidis*	DNA	Neisseriaceae	Humans	TLR9	(45)
*Mycobacterium tuberculosis*	DNA	Mycobacteriaceae	Humans	TLR9, AIM2, cGAS	(46–48)
*Francisella tularensis*	DNA	Francisellaceae	Mammals, birds, amphibians and fish	AIM2	(49)
*Francisella novicida*	DNA	Francisellaceae	Humans	cGAS, p204	(50)
*Streptococcus pneumoniae*	DNA	Streptococcaceae	Humans	AIM2, cGAS	(51, 52)
*Listeria monocytogenes*	DNA	Listeriaceae	Humans and ruminants, etc.	AIM2, IFI16, cGAS	(28, 53)
*Mycobacterium bovis*	DNA	Mycobacteriaceae	Mammals	p204	(54)
*Staphylococcus aureus*	DNA	Staphylococcaceae	Humans, dogs, cats, cows and chickens	p204, AIM2	(55, 56)
*Aspergillus fumigatus*	DNA	Trichocomaceae	Humans	AIM2, TLR9	(57, 58)

AIM2, Absent in melanoma 2; cGAS, cyclic GMP-AMP synthase; DAI, DNA-dependent activator of IRFs; DDX41, DEAD (Asp-Glu-Ala-Asp) box polypeptide 41; DHX9, DEAD/H (Asp-Glu-Ala-Asp/His) box polypeptide 9; DHX36, DEAD/H (Asp-Glu-Ala-Asp/His) box polypeptide 36; DHX60, DEAD/H (Asp-Glu-Ala-Asp/His) box polypeptide 60; IFI16, IFN-gamma-inducible protein 16; DNA-PK, DNA-dependent protein kinase; RNA pol III, RNA polymerase III; MRE-11, meiotic recombination 11 homolog A; TLR9, Toll-like receptor 9.

## Sources of Cytotoxic DNA

The cytosol of eukaryotic cells is deprived of DNA under physiological conditions; nevertheless, multiple factors can contribute to the accumulation of ss or dsDNA in the cytosol, for example, infection by DNA viruses (59), infection by retroviruses which carry out their transcription in the cytosol through the action of viral retro-transcriptase (60), endosomal escape of bacteria (59), activation of regulated cell death (RCD) pathways which results in mitochondrial rupture and consequent release of mitochondrial DNA (mtDNA) into the cytosol (9, 61), reactivation of endogenous retroviral sequences (10), genetic mutations in affecting the activity of the nucleases (12), the formation of micronuclei due to mitotic defects (11, 62, 63), DNA damage following radiation therapy (64) and cytosolic DNA accumulation following phagocytosis, micropinocytosis or uptake of DNA-rich exosomes (65, 66). Hence, there is a continuous risk of cytosolic accumulation of ectopic DNA in both normal and malignant cells, which needs to clear off efficiently to maintain the normal functions of the cells.

## DNA Sensors

DNA sensors are DNA-binding proteins that are component of the innate immune system which are capable of detecting perturbations in DNA homeostasis of the cell and activate the intracellular signaling cascades of the innate immune system as a response (67). DNA sensors can induce a broad range of innate immune responses, and such responses are of particular importance during viral infection when elicitation of type I IFNs is a key immune response that works in a paracrine and autocrine way to confer an anti-viral immunity to the host (4). Type I IFNs, which are induced during the anti-viral immunity, control the viruses in infected cells and restrict their spread to neighboring cells. DNA sensors not only induce type I IFNs but also induce programmed cell death as an innate immune response to the infection. For example, cGAS-STING and TLR9 can induce apoptosis, while IFI16 and AIM2 can induce pyroptosis (68). Although our understanding of the molecular and structural features of DNA sensors has increased significantly over the last few years, however, it is still unclear how various DNA sensing systems are allocated to various locations within the cells and how they cooperate. Differentiating viral and self DNA is very crucial for the host to launch suitable innate responses against viral infections. Based on current knowledge, the signaling specificity of DNA sensors is attributed to various factors such as (i) length, 3D structure and sequence of cytotoxic DNA (8, 69, 70); (ii) subcellular localization of DNA molecules (71); (iii) methylation status of DNA (68) and (iv) association of histones and non-histone chromatin-binding proteins with cytotoxic DNA molecules (8, 71). How the actual source of cytotoxic DNA and each of the factors mentioned above impact the activity of various DNA sensors yet remain to be fully explored.

**Figure 1:**
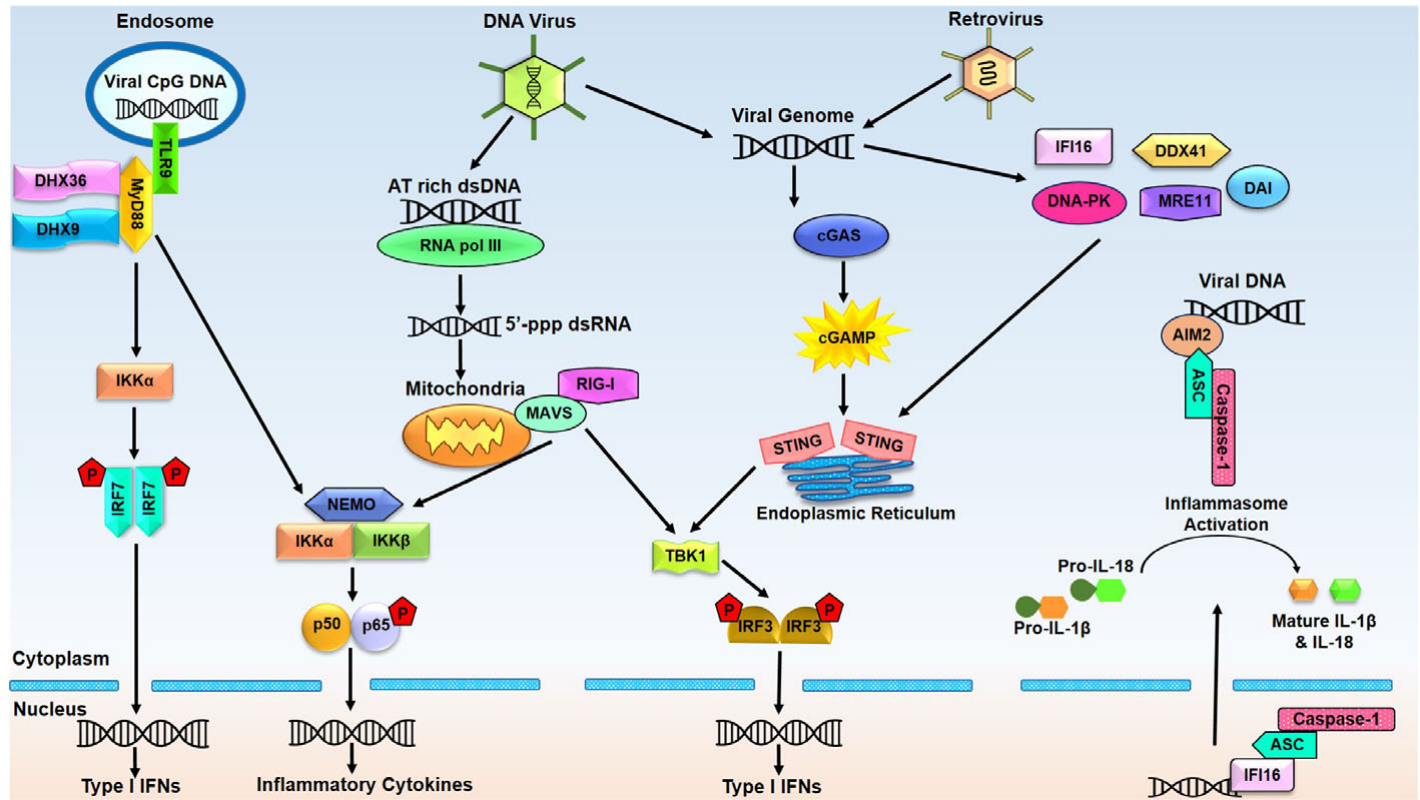
Endosomal and cytosolic DNA sensors and their related signaling pathways. Endosomal DNA sensor TLR9 recognizes the CpG DNA of viral origin and recruits MyD88 leading to activated IRF7 and NF-κB, which mediate induction of type I interferons (IFNs) and inflammatory cytokines. RNA pol III transcribes AT-rich double-stranded DNA (dsDNA) into 5’-triphosphate double-stranded RNA (5’-ppp-dsRNA), leading to the activation of the RIG-I-MAVS signaling pathway. Viral or bacterial DNA can also be detected by cGAS and other putative DNA sensors, all reported to activate the endoplasmic reticulum residing adaptor protein STING. STING travels from the endoplasmic reticulum to the Golgi complex for TBK1-IRF3 and NF-κB activation, triggering the production of type I IFN and inflammatory cytokines. AIM2 and IFI16 detect the viral DNA and respond by forming inflammasome by recruiting ASC and caspase-1 in the cytoplasm and nucleus. Active inflammasome leads to proteolytic cleavage of pro-IL-β and pro-IL-18 to produce mature cytokines. (AIM2, absent in melanoma 2; cGAMP, cyclic GMP-AMP; cGAS, cyclic GMP-AMP synthase; DAI, DNA-dependent activator of IRFs; DDX41, DEAD-box polypeptide 41; DHX9, DEAH-Box Helicase 9; DHX36, DEAH-Box Helicase 36; DNA-PK, DNA-dependent protein kinase; ER, endoplasmic reticulum; IFI16, interferon gamma-inducible protein 16; IFN, interferon; IRF3, Interferon regulatory factor 3; IRF7, Interferon regulatory factor 7; IL-1β, Interleukin-1β; IL-18, Interleukin-18; MAVS, Mitochondrial antiviral-signaling protein; MRE11, meiotic recombination 11 homolog A; NF-κB, Nuclear factor-κB; NEMO, NF-kappa-B essential modulator; RIG-I, Retinoic acid-inducible gene I; STING, Stimulator of interferon genes; TBK-1, TANK-binding kinase 1).

There exist two broad categories of innate immune DNA sensors based on their expression pattern and subcellular localization. The first category comprises endosomal DNA sensors, such as members of the TLR family. Located in the endosomal membrane of many immune cells such as macrophages, dendritic cells (DCs), and B cells, these TLRs monitor the lumen of lysosomes and endosomes for the presence of cytotoxic DNA, e.g., bacterial and viral DNA. The second category accounts for the cytosolic DNA sensors that detect cytoplasmic nucleic acids in virtually all types of cells. **Figure 1** depicts the signaling cascades and resultant immune responses which are triggered by various DNA sensor.

## Endosomal DNA Sensors

### TLR DNA Sensors

Members of the TLR family have the propensity to detect a range of microbial products such as DNA, RNA, and microbial surface molecules. TLRs are type I transmembrane receptors, and they harbor extracellular leucine-rich repeats (LRRs), a transmembrane domain, and a Toll/IL-1 receptor (TIR) domain, which can transduce signals to downstream adaptor molecules such as TIR-domain-containing adapter-inducing interferon-β (TRIF) and myeloid differentiation primary response gene 88 (MyD88) which bring about NF-κB activation. In humans, 10 members of TLRs have been identified, of which five members TLR3, TLR7, TLR8, TLR9, and TLR13 are involved in recognition of pathogenic nucleic acids. These receptors function by utilizing two signaling pathways: TLR7, TLR8, TLR9, and TLR13 mediate the activation of MyD88, while TLR3 activates TRIF (72–74). At present, TLR3, TLR7, TLR8, and TLR9 have been structurally characterized.

### TLR9

TLR9 is the only known endosomal localized DNA sensor and was the first reported PPR to detect DNA (68). TLR9 is highly expressed in both plasmacytoid dendritic cells (pDCs) and B cells, and senses un-methylated cytosine–phosphate–guanosine (CpG) motif-containing DNA of viral and bacterial genomes (68, 75, 76) and results in the induction of IFN-α, IFN-λ, many chemokines and cytokines (13, 77–79). The CpG motifs in mammals are methylated at the cytosine base (80), while the bacterial and viral CpG sites are un-methylated; therefore, TLR9 can distinguish between self and non-self to prevent unwanted immune reactivity (81). TLR9 has been reported to detect the DNA of herpes simplex viruses 1 and 2 (HSV-1 and HSV-2), herpes papillomavirus (HPV), varicella-zoster virus (VZV), Merkel cell polyomavirus, cytomegalovirus (CMV), Kaposi’s sarcoma-associated herpesvirus (KSHV), ectromelia virus (ECTV) and Epstein-Barr virus (EBV) (29, 32, 33, 36, 37, 82). Additionally, a role for TLR9 in the detection of HIV has also been suggested (38).

In unstimulated pDCs, TLR9 is found associated with the endoplasmic reticulum (ER) in its inactive form. Upon the presence of CpG DNA, TLR9 is trafficked to the lysosomes by the action of 12-membrane-spanning ER protein UNC93B, which interacts with TLR9 directly (83). In endolysosomal compartments, the proteolytic cleavage of TLR9 in response to the presence of CpG DNA converts it into active processed form (84). Clathrin-dependent endocytic pathways internalize CpG DNA, which is then translocated to the lysosomes, interacting with active TLR9. It is still ambiguous how TLR9 is triggered to translocate from ER to CpG containing lysosomes. After recognizing CpG DNA, TLR9 interacts with its adaptor protein MyD88, which contains a death domain and a TIR domain (85). MyD88 further interacts with IL-1R associated kinase 1 (IRAK-1), IRAK-4, and IRF-7, which subsequently induces TNF receptor-associated factor 3 (TRAF3) and TRAF6 recruitment, activating the transforming growth factor β-activated kinase 1 (TAK1), mitogen-activated protein kinase (MAPK), and NF-κB ultimately inducing the inflammatory cytokines (85).

**Figure 2:**
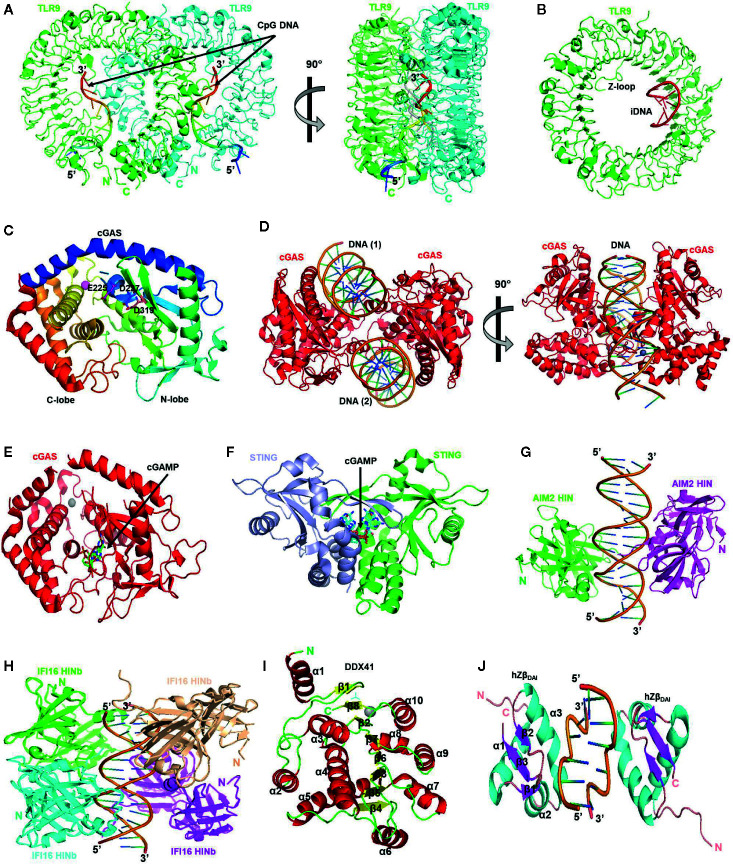
The structures of endosomal and cytosolic DNA sensors. **(A)** TLR9/CpG DNA complex. CpG DNA binding induces the dimerization of TLR9. Two TLR9 molecules are shown in green and cyan colors (PDB code 3WPC) **(B)** TLR9/iDNA complex. iDNA shown in red color forms a stem-loop structure that occupies the interior of ring-shaped TLR9 shown in green color (PDB code 3WPD). **(C)** The overall structure of apo-form of human cGAS. The catalytic residues are shown in the sticks (PDB code 4MKP). **(D)** The structure of human cGAS catalytic domain bound to 18 bp dsDNA. DNA binds to two distinct positively charged surfaces of cGAS, inducing dimerization and conformational rearrangement of cGAS active site (PDB code 4O6A). **(E)** The overall structure of cGAS in complex with 2’3’-cGAMP (PDB code 6MJX). **(F)** The structure of STING bound with cGAMP which is shown as sticks (PDB Code 5CFP). **(G)** The structure of the AIM2 HIN : DNA. HIN domains are represented as green- and magenta-colored ribbons with DNA positioned between them (PDB code 3RN2). **(H)** The structure of the IFI16 HINb: DNA complex is shown as green, cyan, wheat, and magenta ribbons for each HINb domain and orange ribbon for the dsDNA (PDB code 3RNU). **(I)** A ribbon representation of the DEAD domain of DDX41 with secondary structural elements labeled. Helix, sheet, and loop are colored in red, yellow, and green, respectively (PDB code 5H1Y). **(J)** The overall structure of the hZβ_DAI_/Z-DNA complex. The protein and DNA are drawn as a ribbon diagram. The N and C termini, the secondary structure elements of hZβ_DAI_, and 5′ and 3′ of DNA are labeled. Helix, sheet, and loop are colored in cyan, magenta, and light pink, respectively (PDB code 3EYI). All the images in the figure were drawn by PyMOL molecular graphics system (v1.7.4.0) by using the mentioned PDB IDs which were obtained from Protein Data Bank (https://www.rcsb.org/).

Like other TLRs, TLR9 also contains an extracellular LRR domain carrying out ligand recognition, a transmembrane domain, and a cytoplasmic TIR domain that interacts with adaptor proteins and initiates downstream signaling cascades. TLR9 contains 26 LRRs arranged in a ring-shaped structure maintained by multiple interactions (86). A long inserted loop called Z-loop containing about 40 amino acid residues is present in TLR9, whose proteolytic cleavage in endolysosomes is reported to be necessary for the generation of mature functional TLR9. This cleavage also prevents undesired activation of the receptor by the cellular DNA (87). At present, three types of crystal structures of TLR9 are available: unliganded TLR9, CpG-DNA bound TLR9, and inhibitory DNA (iDNA) bound TLR9 (86). These structures have conferred crucial information on the functional mechanism and signaling activities of TLR9. Based on these structures, the activation mechanism of TLR9 has been proposed, which describes that inactive TLR9 is present as a monomer and it dimerizes upon ligand binding to attain an active “m” shaped structure, in which two TLR9 protomers closely position their C-terminal regions as shown in **Figure 2A**. The dimerization of LRR domain regions also induces TIR domain dimerization, which leads to the resultant recruitment of adaptor proteins. The unliganded TLR9 is present in a ring-shaped monomeric form in both solution and crystal and manifests the inactive form of TLR9 (88). It has been validated through ultracentrifugation, and gel-filtration analysis that cleaved TLR9 dimerizes upon CpG DNA binding (86). Although TLR9 having intact Z-loop has also been shown to bind with CpG DNA, but this binding does not induce dimerization of TLR9; therefore, Z-loop processing, if not necessary for binding with DNA, is essential for mediating the CpG-DNA-induced dimerization of TLR9 (86). In ligand-bound TLR9, a 2:2 complex of TLR9 and CpG-DNA is formed in which CpG-DNA is wedged between the two TLR9 protomers and stabilizes the structure as shown in **Figure 2A**. In this structure, two C-terminals of TLR9 dimer are located at a proximity of approximately 30 Å from each other. The CpG binding groove formed by the LRR-NT, LLR1, and LLR2 is located at the N-terminus of interface 1 of TLR9, and multiple interactions are formed between cytosine and guanosine of CpG motif and TLR9 in the binding groove. The flanking regions of CpG dinucleotide also contribute the binding, interface 2 of TLR9 involves in recognizing the phosphate backbone of the CpG DNA, and histidine residues in interface 2 establish electrostatic interactions with phosphate groups present in the DNA backbone (86). The binding of TLR9 to CpG DNA is pH-dependent (89), and under acidic conditions, the binding affinity is stronger (86). The crystal structure of the TLR9-iDNA complex depicts that iDNA is present as a stem-loop structure formed through intramolecular base pairing, and it engages the interior of the TLR9 ring structure (**Figure 2B**), and in contrast to the TLR9–CpG–DNA complex, which exhibits 2:2 stoichiometry, TLR9-iDNA is a monomer (86).

## DNA Sensors in the Cytosol

After endocytosis, many DNA viruses pass through the cytoplasm to reach the nucleus where they release their genomic material. Viral capsid protects the DNA genomes and is not discarded until the viral DNA is injected into the nucleus; therefore, it is worthy of questioning how DNA sensors in the cytosol detect viral DNA under physiological conditions. This question is easier to answer for viruses like smallpox, which replicates in the cytoplasm (90), and polyomavirus simian virus 40, whose capsid is dissembled in the ER and its genomic DNA is released into the cytoplasm (91). Hence, such viruses can trigger the DNA sensing pathways in the cytosol. Nonetheless, many viruses such as herpesviruses expose their DNA only in the nucleus; therefore, there must exist some mechanisms that leak their DNA into the cytoplasm. One explanation for herpesviruses DNA is that it can be sourced from the defective virion particles in the cytoplasm and is ultimately detected by the cytosolic DNA sensors. In HCMV and HSV-1, ubiquitination can label the capsid for proteasomal degradation in the macrophages, leading to the release of their DNA into the cytoplasm (14). Cellular stress-dependent leakage of mtDNA can also occur in the case of herpesviruses, which can lead to the activation of the cGAS-STING pathway (92).

Unlike endosomal sensors for viral sensing, which are limited to TLRs, cytosolic DNA sensors present an array of different PRRs that sense viral nucleic acids and lead to the production of either type I interferons/inflammatory cytokines or caspase 1-dependent secretion of IL-1β. Since type I IFN production is the major anti-viral defense strategy employed by the host, it is the main outcome of DNA sensing in the cytosol. Multiple cytosolic DNA receptors have been identified through intensive investigation of past years such as DAI, RNA polymerase III, cGAS, AIM2, and IFI16, which results in type I IFN production by converging at a common pathway, STING-pathway (59). STING is a transmembrane protein expressed by the outer mitochondrial membrane and ER, and it relocalizes with TANK-binding kinase 1 (TBK1), which executes phosphorylation activation of IRF3 and IRF7 (59, 93). The STING-TBK1 axis is pivotal for driving interferon responses and host resistance against DNA viral infections (59). In the next section, we will discuss major anti-viral cytosolic DNA sensors.

### cGAS-STING Pathway

Cyclic GMP-AMP synthase (cGAS) is a DNA-sensing nucleotidyl transferase enzyme that is a member of the nucleotidyltransferase (NTase) family and functions as a cytosolic DNA sensor (41, 94). cGAS is known to recognize various viruses such as DNA viruses, including vaccinia virus, HSV1 and HSV2, cytomegalovirus, adenoviruses, human papillomavirus, and murine gammaherpesvirus 68, which are counteracted by type I IFNs through cGAS–STING pathway (15). Retroviruses such as murine leukemia virus, simian immunodeficiency virus (SIV), human immunodeficiency virus (HIV), West Nile virus, vesicular stomatitis virus (VSV), and Dengue virus have also been reported to be detected by cGAS (40, 41). Besides, it can also sense Gram-positive and Gram-negative bacteria. It is activated by direct binding with DNA, and this binding induces liquid–liquid phase separation to produce liquid droplets acting as a microreactor where the concentration of cGAS is enhanced to increase the synthesis of cyclic GMP-AMP (cGAMP) utilizing ATP and GTP (94, 95). cGAMP has unique mixed phosphodiester linkages between the 2′-hydroxyl group of GMP and the 5′-phosphate of AMP, and also between the 3′-hydroxyl group of AMP and the 5′-phosphate of GMP, forming a unique 2′3′-cGAMP isomer (96, 97). cGAMP’s binding to STING yields dimers, tetramers, and higher-order oligomers of STING (98) and activates the STING to produce type I IFNs and NF-κB-dependent proinflammatory cytokines (94).

cGAS can be activated by both self and foreign DNA to induce conformational changes in its structure that are necessary for its enzymatic activity. It can bind to DNA of ~20 bp, but longer dsDNAs of >45 bp result in a ladder-like structure of cGAS dimers, which are more stable and have stronger enzymatic activity (99, 100). The binding affinity of cGAS to dsDNA and ssDNA is *K*d ∼87.6 nM and *K*d ∼1.5 μM, respectively (101). Many groups have solved the structure of cGAS alone or DNA-bound cGAS (99–104) (**Figures 2C-E**), which provides significant insights about mechanistic aspects of cGAS activation by DNA binding and its enzymatic activity. A substantial conformational change is observed in cGAS upon DNA binding, which induces dimerization and makes its catalytic pocket accessible. The catalytic domain of cGAS possesses a two-lobed structure in which N-lobe exhibits canonical NTase fold while a tight five-helix bundle is present at C-lobe. A deep groove between these two lobes contains the active site, which has three catalytic residues, glutamate 225, aspartate 227, and aspartate 319, crucial for the enzymatic activity of cGAS because their mutations have been shown to abrogate enzymatic activity (99). The C-terminal region of cGAS contains a conserved zinc ribbon domain, essential for its activity (101, 102). In cGAS dimer, hydrogen bonding between the residues of the zinc-binding loop joins the two molecules of cGAS. cGAS is inactive before DNA binding since its active site presents a scrambled structure, and the NTase domain is destabilized (99). The structure of porcine and mouse cGAS:dsDNA complexes (102, 103) shows that cGAS and dsDNA bind with a 1:1 stoichiometry, and interaction occurs *via* the single binding site. However, two other studies have reported that a 2:2 complex in which each cGAS molecule binds to two dsDNA molecules *via* two binding sites (**Figure 2D**), one of which is the same as reported by previous studies (102, 103), while one binding site is new (99, 100). Both DNA binding sites contain multiple positively charged residues and have shape and charge complementarity with dsDNA (99). After activation of cGAS, a two-step catalytic reaction mediates the formation of cGAMP, and an intermediate pppGpA is formed, and then cyclization of this intermediate yields cGAMP (103). When cGAMP binds with STING, it leads to a conformational change by which two wings of STING are brought to each other in juxtaposition, and the ligand is buried deep in the binding pocket (**Figure 2F**). The binding pocket shows a top lid consists of four antiparallel β-sheet strands, which confer a close confirmation to the structure. A rotation of 180° is observed in ligand-binding pocket upon cGAMP and STING binding, which results from side-by-side packing of STING dimers yielding STING oligomers (105).

Presence of cGAS in the nucleus has been reported by multiple studies (106–108) however, recently it is proposed that tight tethering of chromatin to the cGAS suppresses autoreactivity to self-DNA in the nucleus. The structure of the cGAS catalytic domain bound to a nucleosome has been resolved by many groups, which reveals that cGAS inhibition in the nucleus is mediated by interaction through histone 2A–2B but not through nucleosomal DNA binding. The interaction between cGAS and histone embeds the cGAS DNA-binding site B, and prevents the formation of active cGAS dimers (108–110). Kujirai et al. has reported a cryo-electron microscopy structure with two cGAS molecules bridging two nucleosome core particle (NCP). This configuration shows that all three known cGAS DNA binding sites that are required for cGAS activation become inaccessible, and cGAS dimerization is also inhibited (111). Another structure by Boyer et al. reported the structure of cGAS bound to a single nucleosome. This binding sterically abrogates cGAS oligomerization required to yield functionally active 2:2 cGAS–dsDNA complex (112). These recent findings have provided important information that how cGAS is maintained in an inhibited state in the nucleus.

STING contains four transmembrane helices (TM1–TM4), one folded soluble domain previously assigned as TM5, and a large cytosolic domain (amino acids 173–379) (113, 114). STING is kept in the ER through its binding to Ca^2+^ sensor stromal interaction molecule 1 (STIM1) (115); however, it’s binding to cGAMP mediates its trafficking from ER to ER–Golgi intermediate compartment (ERGIC) and the Golgi apparatus by the action of cytoplasmic coat protein complex II (COPII) and ADP-ribosylation factor (ARF) GTPases (116). The palmitoylation of STING takes place in the Golgi apparatus, which is crucial for its activation (117). After translocation to the Golgi apparatus, STING binds with TBK1, which phosphorylates the C-terminal tail region of STING, a docking site for IRF3. TBK1 also phosphorylates IRF3 inducing its activation (104) and activated IRF3 dimerizes and translocate to the nucleus to regulate the transcription of interferon-β (IFNβ) (118), which activates heterodimeric receptor complex comprising IFNα receptor 1 (IFNAR1) and IFNAR2, which further activates the Janus kinase (JAK)-signal transducer and activator of transcription (STAT) signaling pathway to incite the transcription of several ISGs whose protein products ultimately block viral replication, assembly, and release (119). Downstream of the cGAS-STING pathway, programmed cell death, mainly apoptosis, can be activated. Furthermore, the cGAS-STING pathway can induce necroptosis, as well (120).

### STING as DNA Sensor

STING has been demonstrated to bind DNA directly, but we still need to fully disclose the physiological relevance of DNA binding by STING (121). A study has reported that amino acids 181–379 in the C-terminal of STING could bind the dsDNA without any stipulation from other proteins; nonetheless, STING bound to dsDNA with only *K*d ∼200–300 μM affinity, which is significantly lower than the binding affinity of the cGAS to DNA (*K*d ∼88 nM). Furthermore, ectopic expression of STING in HEK293T cells, which are deprived of endogenous STING, did not produce IFNβ in response to dsDNA, suggesting that STING cannot execute DNA sensing in cells (94, 122). Therefore, future studies are needed to verify if STING can act as a DNA sensor.

STING polymorphism is suggested to be involved in the pathogenesis of Coronavirus disease 2019 (COVID-19). No data is currently available to demine if COVID-19 alters STING activation during early infection; however, during the second phase of infection, an excessive amount of damaged host DNA activates the STING, which ultimately causes cytokine storm, a characteristic feature of COVID-19 (123).

### PYHIN Family Members

PYHIN protein family (pyrin and HIN200 domain-containing proteins, also known as p200 or HIN200 proteins) have been associated with recognizing both microbial and self DNA, resulting in a wide range of innate immune responses. The characteristic features of most family members are the presence of pyrin domain (PYD) at N-terminal capable of mediating protein-protein interactions and one or two C-terminal HIN200 domains, which carry out DNA binding (124). The human genome has been reported to encode 4 PYHIN proteins (124), out of which two proteins, absent in melanoma 2 (AIM2) and IFN-γ inducible 16 (IFI16), are known DNA sensors and have the propensity to execute DNA-induced innate immune responses (16, 125, 126). Structures of PYHIN proteins coordinate with their proposed role as DNA PRRs, and members AIM2, IFI16, and murine protein p204 are now designated to a new family of PRRs termed as AIM2-like receptors (ALRs) (127).

### AIM2

AIM2 is mainly expressed in intestinal epithelial cells, keratinocytes, and monocytic lineage (126, 128) and can detect DNA from diverse sources such as self-DNA, bacterial and viral DNA (125, 129, 130). AIM2 is reported to detect vaccinia virus and mouse cytomegalovirus (28). AIM2 contains an N-terminal PYD domain, C-terminal HIN200 domain, which is positively charged and binds with negatively charged DNA. DNA sensing by AIM2 results in the assembly of inflammasome, which is a supramolecular multi-protein complex. The PYD domain of AIM2 establishes interaction with the PYD domain of the adaptor protein of inflammasome known as an apoptosis-associated speck-like protein containing a carboxy-terminal CARD (ASC), while the CARD domain of ASC associates with the CARD domain of pro-caspase-1, leading to the assembly of activated AIM2 inflammasome (126). The autocatalytic cleavage of pro-caspase-1 generates caspase-1, which converts pro-IL-18 and pro-IL-1β into their active forms, which, in turn, mediate downstream inflammatory responses and pyroptosis. AIM2 inflammasome is also known to induce apoptosis (131). AIM2 inflammasome is entirely indispensable for type I IFNs production in response to dsDNA (132, 133), while it is essential to produce active caspase-1 to induce inflammatory responses. This fact underscores that cells use different mechanisms to execute innate immune responses against cytotoxic DNA. Currently, two structures of AIM2^PYD^ are available, one harbors an N-terminal MBP tag, while the other contains surface mutations and these structures reveal that AIM2^PYD^ adopts a six helical bundle shape, which is a characteristic feature of the death domain superfamily (134, 135). It has been proposed that AIM2^PYD^ domain is sequestered by the AIM2^HIN^ domain through intramolecular interactions during the resting state of AIM2; however, upon dsDNA binding, AIM2^PYD^ is displaced from the association of AIM2^HIN^ so that it can interact with PYD domain of ASC (134). However, a later study by Sohn and colleagues reported that acid patch mutant of AIM2^PYD^, which had impaired binding with AIM2^HIN^, also presented loose binding between dsDNA and AIM2^HIN^, thus, ruling out the previously described inhibitory role of AIM2^PYD^.

Furthermore, after reaching a certain threshold concentration, full-length AIM2 was able to self-associate with DNA; therefore, DNA serves as a one-dimension ruler upon which AIM2 clusters itself and increases its local concentration (136). The structure of DNA-bound AIM2^HIN^ domains (**Figure 2G**) reveals that the molecular basis of DNA sensing by AIM2 is sequence-independent because all the interactions of AIM2^HIN^ take place with the phosphate backbone of the dsDNA, not with the individual DNA basis (137). In X-ray crystallographic structure, both strands of B-form DNA are bound by the HIN domain through electrostatic interactions between arginine and lysine of HIN, and sugar and phosphate groups of DNA backbone (134, 137) (**Figure 2G**).

Full activation of AIM2 in cells requires ~80 bp of dsDNA, while isolated AIM2^HIN^ associates with 20-bp dsDNA of ~30 nM affinity, although the footprint of one AIM2^HIN^ is 8–9 bp (137). Even when DNA is present in excess amount, AIM2^HIN^ and AIM2^FL^ both clustered upon the same DNA molecule (136, 138). AIM2^PYD^ does not bind with DNA, but it is involved in the clustering of AIM2^HIN^ on DNA molecules. Moreover, the weak interactions among AIM2^HIN^ protamers also contribute to DNA clustering because mutating the residues involved in HIN: HIN interactions in AIM2^HIN^ and AIM2^FL^ also diminished their cooperative binding with dsDNA (136). Cryo-EM structure has revealed that binding of multiple AIM2 molecules on the same DNA enhances the local concentration of AIM2^PYD^, which then interacts with each other to produce long helical filaments with the core filament being a right-handed one-start hollow filament having an inner diameter of ~20 Å and an outer diameter of ~90 Å (138). ASC^PYD^ subunits assemble to form filaments using AIM2 as a nucleating platform, and it has been demonstrated that AIM2^FL^ + dsDNA and AIM2^PYD^ both promoted the filament formation by ASC^PYD^ subunits (139). Negative stain EM spectra and crystal structure both have reported similarities in subunit organization and diameter of AIM2^PYD^ and ASC^PYD^ (136, 139).

### IFI16

The first cytosolic DNA sensor to be reported was IFN γ-inducible protein 16 (IFI16), which induces innate immune responses against ss and ds intracellular cytotoxic DNA (16, 140). It has been reported to sense the DNA of many viruses such as herpesviruses (17, 141), Kaposi’s sarcoma herpesvirus (KSHV), cytomegalovirus, and Epstein–Barr virus to mediate STING-dependent IFN-β responses (14, 16, 34). Located predominantly in the nucleus and in small fractions in the cytoplasm, IFI16 can function to activate both type I IFN responses and functional ASC- and CASP1-containing inflammasome (17, 142). For example, during KSHV infection, after recognizing the viral dsDNA, IFI16 forms the AIM2-independent inflammasome complex, which is then transported to the cytoplasm (17); however, details of inflammasome formation by IFI16 are still not fully clear. Furthermore, during HIV infection, IFI16 mediates caspase-1 activation resulting in pyroptosis (143). In contrast, HSV-1 infection leads to IFI16-STING mediated production of IFNβ (16). In macrophages and keratinocytes, IFI16 has been indicated to activate the catalytic activity of cGAS in addition to employing the effectors of STING (144).

The murine PYHIN protein p204 is an orthologue of IFI16 and was crucial for HSV-1 and DNA-induced activation of transcription factor and expression of IFNβ in a macrophage cell line of the mouse (16). It comprises two HIN domains named as HINa and HINb, and contains N-terminal PYD domain that can establish homotypic interactions with other PYD-containing proteins to form higher complexes (145). Due to similarity in domains structure, p204 is suggested to perform similar functions as IFI16, however, further evidience is required to fully establish its role.

IFI16 contains one N-terminal PYD domain, and two tandem HIN200 domains termed as HINa and HINb. The nature of the PYD domain of IFI16 differs from AIM2; thus, it may use a different mechanism for inflammasome assembly as compared to AIM2. The crystal structure of the HIN domain revealed two interlinked oligonucleotide/oligosaccharide binding (OB) fold domains (146, 147). The structure of both DNA-bound HINa and DNA-bound HINb is available now (137, 148) **(** **Figure 2H**) which revealed that IFI16 binds to dsDNA in a cooperative and length-dependent manner (142, 149), and scans the dsDNA in one-dimension utilizing its HIN domains (142). HIN domains of IFI16 bind with both ss and dsDNA mainly through electrostatic interactions (16) with the same affinity because dsDNA is recognized as two single strands by HIN domains (148). Although both HINa and HINb can bind to the DNA, they have different affinities for DNA binding (16), and have different DNA binding surfaces (148).

The HIN domains of IFI16 have been proposed to use two distinct modes of DNA binding. The first mode represents AIM2-like DNA binding, in which the linker joining the two OB folds is used as a tether to bind to DNA (137), while the second mode is like p202 HINa binding to DNA in which loops from OB1 and OB2 folds are utilized for DNA binding (150). It has also been suggested that these two distinct modes of DNA binding mediate different immune responses. IFI16 is also implicated to participate in DNA damage response pathways (17); therefore, it is also possible that it can bind with nicks, gaps, and ends of damaged DNA resulting in the initiation of immune responses. Evidence for this feature comes from the ability of the HIN1 domain to recognize different DNA topologies (148). For most of the *in vitro* tested DNA, HINa domain can form complex with DNA relatively faster than HINb, while HINb binds GC-rich DNA more tightly than HINa. One domain of HINb interacts with both strands of DNA, while one domain of HINa binds only one strand of DNA (148). As stated previously, the PYD domain of IFI16 is different from PYD domains of other PYHIN family members; therefore, we need future studies to elucidate the exact mechanism that how the PYD domain of IFI16 interacts with STING to mediate IFN production.

### DExD/H-Box Helicase Family Members (DHX9, DHX36, DDX41, DDX60)

DExD/H-Box helicase family has many RNA and DNA helicases involved in DNA-mediated production of type I IFNs. Two subgroups are present in this family, which are the DEAH-box helicases (DHX) and the DEAD-box helicases (DDX) (18, 19). DEAD/H (Asp-Glu-Ala-Asp/His) box polypeptide 9 (DHX9) and DHX36 are involved in the sensing of dsRNA in myeloid DCs and CpG-rich DNA in human pDCs. DHX9 regulates TNF-α expression and induces the activation of NF-κB through MyD88 in human pDCs, whereas DHX36 induces the production of IFN-α and IRF7 activation through MyD88 (19). DDX60 can sense both dsRNA and dsDNA and mediates the expression of CXCL10 and IFN-β. It also augments signals from RIG-I and MDA5 (42).

### DDX41

DEAD (Asp-Glu-Ala-Asp) box polypeptide 41 (DDX41) is a cytoplasmic DNA sensor and has been reported to detect the DNA of HSV-1 and adenovirus in myeloid DC and murine bone marrow-derived DC. It can induce type I IFN response through STING-TBK1 signaling after sensing DNA through its DEAD domain. Upon limiting the basal expression of IFI16 *in vitro*, DDX41 served as the initial cytoplasmic DNA sensor and induced the IFN expression; thus, it can be deduced that the expression pattern of different DNA sensors may define their innate response pattern (18).

DDX41 comprises a disordered N-terminal region, a helicase domain, and a DEAD domain. These two domains are conserved among the DEAD-box family members, and they contain multiple conserved motifs, e.g., motif I and Q motif, which are crucial for ATP binding (151). The currently available crystal structure of DDX41 is based on truncated hDDX4 protein and reveals α/β fold found in other DEAD-box family proteins. There are ten α-helices (α1–α10) and a β-sheet organized by eight β-strands (β1–β8) in the overall structure (**Figure 2I**). Helices α1-α5 are present on one side of the β-sheet, whereas helices α6–α10 are positioned on the other side (152). The DEAD domain’s crystal structure contains motif Q, P-loop, motif Ia, motif Ib motif II, and motif III positioned at either β-strand-loop or helix loop transitions. Nucleotide-binding is associated with the P-loop (152). Binding with the dsDNA facilitates the interaction of DDX41 with STING, which ultimately induces type I IFN production (148). The dsDNA-bound DEAD domain’s docking model suggested that the DNA-binding site involves arginine 267, lysine 304, tyrosine 364, and lysine 381 present at the C-terminal region (151).

Although DDX41 is reported as a DNA sensor by multiple studies, some studies have also reported that RNAi induced depletion of DDX41 resulted in little effect on the induction of IFN-β upon stimulation with DNA virus infection or DNA (121, 153, 154); therefore, further research is indispensable to clarify the exact role of DDX41 as a DNA sensor.

### RNA Polymerase III

RNA polymerase III (RNA pol III) serves as a cytosolic dsDNA sensor through produced RNA and transduces signals for RIG-I and MAVS signaling pathways (20, 30). Initially, it was very puzzling that how poly (dA:dT) in some human cell lines could induce IFN-β production through RIG-I/MAVS signaling pathways; however, subsequent research resolved this conundrum by demonstrating that transfected poly (dA:dT) is converted into RNA containing 5’-triphosphate and double-stranded secondary structures by the action of RNA pol III which serves as bona fide trigger of RIG-I (20, 30). This feature gives the host advantage of utilizing the RIG-I-MAVS pathway to detect DNA viruses and bacteria. RNA pol III mediates the synthesis of IFN-inducing small RNA from the DNA of adenovirus in murine bone marrow-derived DCs (155), and inhibition of RNA pol III affected late immune responses during adenovirus infection in murine RAW267.4 cells (156). Although RNA pol III was shown to respond to HSV-1 infection in mouse macrophages (20), the results were challenged by later studies, showing that IFN and cytokine expression are RNA pol III independent in both human and mouse macrophages (16, 157). Nonetheless, recently it was demonstrated that mutations in RNA pol III during VZV infection in children resulted in reduced IFN production, which could not be compensated by other DNA sensors such as cGAS, DDX41, and IFI16 (24). It can be anticipated that future studies will further elaborate on the role of RNA pol III as a DNA sensor in the innate immune responses.

### DAI

DNA-dependent activator of IRFs (DAI, also termed as ZBP1 or DLM1) was the first putative DNA sensor identified by Takaoka et al. and was found to mediate IRF3 activation through TBK1 leading to type I IFNs production (21). Overexpression of DAI resulted in elevated DNA-induced synthesis of type I IFNs, while its inhibition through RNAi suppressed IFN induction in L929 cells. Despite first reports designating DAI as a cytosolic sensor of viral DNA, later studies using DAI-deficient mouse embryonic fibroblasts and mice reported them to induce normal IFN response (158). Therefore, DAI maybe working as an indispensable cytosolic DNA sensor or maybe cell-type specific; nonetheless, future studies are needed to fully decipher its potential as a DNA sensor. N-terminal domain of DAI comprises 2 tandem Z-DNA binding domains (ZBDs or Zα and Zβ) and a third DNA binding region (D3), which binds right-handed B-DNA is present next to the second ZBD. D3 domain has also been shown to bind Z-DNA. C-terminal of DAI interacts with TBK1 after activation (21). The crystal structure of the Zβ domain of human DAI (hZβ_DAI_) reported that it shares the same fold as other ZBDs but opts for a unique binding mode to recognize Z-DNA. In hZβ_DAI,_ a residue in the first β-strand contributes to the binding with the DNA compared to the residues of β-loop in other ZBDs. This structural data also revealed that both ZBDs of DAI could simultaneously bind the DNA and are required for complete B to Z conversion. It can be expected that the binding of both ZBDs to the same dsDNA may assist in DAI’s dimerization (159). The NMR structure of hZβ_DAI_ reports conformation deviations from its crystal structure, such as the β-sheet wing movement, which disengages the β-loop of the wing from the Z-DNA movement of the recognition helix. The N-terminal of α3 recognition helix contains charged residues, which seems important for recognizing both B- and Z-conformations of DNA (160).

### DNA-PK and MRE-11

DNA-dependent protein kinase (DNA-PK) is a protein involved in DNA damage response and implicated in cytosolic DNA sensing. It comprises three subunits, Ku70, Ku80, and the catalytic subunit DNA-PKcs. Affinity pull-down assays in HEK293T cells have revealed the DNA sensing potential of this protein. Mouse embryonic fibroblasts and mice lacking DNA-PKcs exhibited attenuated cytokine production upon stimulation with viral DNA (161). Furthermore, its subunit Ku70 was also reported to induce IFN-λ1 production upon stimulation with cytosolic DNA in HEK293T cells (22). A very recent study has reported that DNA-PK uses the STING-independent DNA sensing pathway (SIDSP) to exert its functions because the DNA-sensing ability of DNA-PK is not impaired in STING-deficient cells (162). We can anticipate that future updates will render important information on the significance of this new signaling axis.

Meiotic recombination 11 homolog A (MRE-11) is also proposed as a cytosolic DNA sensor that activates the STING pathway (163). MRE-11 is implicated in dsDNA break repair, homologous recombination, and telomere length maintenance. This protein possesses 3’ to 5’ exonuclease activity and endonuclease activity and interacts with RAD50 for non-homologous DNA end-joining (164). Cryo-EM structures of the *E. coli* MRE11-Rad50 homolog SbcCD reveals that in the resting state of MRE11, ATP-Rad50 blocks its nuclease domain. When DNA is bound, its nuclease domain is freed, and it assembles a DNA cutting channel to carryout nuclease reaction on the DNA end (165). Future studies are required to disclose the complete details of its DNA sensing function.

## Concluding Remarks and Future Directions

In the last decade, research in the field of innate immune sensing of pathogen-derived nucleic acids has witnessed fruitful progress and disclosed important signaling cascades such as a cGAS-STING pathway for the detection of cytosolic DNA and RLR-MAVS pathway for sensing cytoplasmic RNA. Furthermore, many DNA sensors’ structural information has undoubtedly yielded important data regarding critical events by which these sensors function. These structural data have advanced our understanding of DNA sensors’ regulatory mechanisms, their ligand-binding sites, proteolytic processing, and how they interact and bind DNA. Further updates in this direction are anticipated to elucidate the potential targets for antiviral therapy. Despite the current progress, many crucial questions are still lacking answers. For example, the cellular compartments are guarded by various innate immune receptors to cope with viral infections and given the fact that many viruses replicate in the nucleus, then there must exist receptors for nuclear surveillance, as IFI16 is predominantly located in the nucleus. It needs to be investigated how the nucleus maintains immune surveillance against viruses and which mechanisms are employed.

Furthermore, there is significant redundancy among the cytosolic DNA sensors with multiple sensors contributing to the antiviral immunity; however, we need to decipher the biological importance of this redundancy and crosstalk between them. Besides, the role of inflammasomes in DNA sensing of viruses yet needs to be fully discovered since only a few inflammasomes are known to participate in viral DNA sensing, while for other pathogens such as bacteria, many different inflammasomes are known. Therefore, there is a possibility that viral DNA may be activating some novel yet unknown inflammasomes. Moreover, we lack comprehensive structural data for many DNA sensors, and it is important to understand the complete structural basis of DNA recognition by these sensors, which can point out important targets for drug development. Finally, it will be of immense significance to know if these DNA sensors detect only naked viral DNA or can sense DNA-associated proteins as well.

## Author Contributions

AZ wrote the manuscript. HI proofread and helped with figure illustrations. TJ and BL provided the guidance and revised the manuscript. All authors contributed to the article and approved the submitted version.

## Funding

This work was supported by the Strategic Priority Research Program of the Chinese Academy of Sciences (Grant No. XDB29030104), the National Natural Science Foundation of China (Grant Nos.: 31870731, 31971129 and U1732109), the Fundamental Research Funds for the Central Universities, and the 100 Talents Programme of The Chinese Academy of Sciences. AZ is supported by CAS-TWAS president fellowship. HI is supported by Chinese government scholarship.

## Conflict of Interest

The authors declare that the research was conducted in the absence of any commercial or financial relationships that could be construed as a potential conflict of interest.
